# Combining mass spectrometry and genetic labeling in mice to report TRP channel expression

**DOI:** 10.1016/j.mex.2021.101604

**Published:** 2021-12-14

**Authors:** Philipp Wartenberg, Femke Lux, Kai Busch, Claudia Fecher-Trost, Amanda Wyatt, Veit Flockerzi, Gabriela Krasteva-Christ, Ulrich Boehm, Petra Weissgerber

**Affiliations:** a Department of Experimental and Clinical Pharmacology and Toxicology, Center for Molecular Signaling (PZMS), Saarland University School of Medicine, Homburg, Germany; b Institute of Anatomy and Cell Biology, Saarland University School of Medicine, Homburg, Germany

**Keywords:** TRP expression, Genetic labeling, Mass spectrometry, Immunoprecipitation

## Abstract

Transient receptor potential (TRP) ion channels play important roles in fundamental biological processes throughout the body of humans and mice. TRP channel dysfunction manifests in different disease states, therefore, these channels may represent promising therapeutic targets in treating these conditions. Many TRP channels are expressed in several organs suggesting multiple functions and making it challenging to untangle the systemic pathophysiology of TRP dysfunction. Detailed characterization of the expression pattern of the individual TRP channels throughout the organism is thus essential to interpret data such as those derived from systemic phenotyping of global TRP knockout mice. Murine TRP channel reporter strains enable reliable labeling of TRP expression with a fluorescent marker. Here we present an optimized method to visualize primary TRP-expressing cells with single cell resolution throughout the entire organism. In parallel, we methodically combine systemic gene expression profiling with an adjusted mass spectrometry protocol to document acute protein levels in selected organs of interest. The TRP protein expression data are then correlated with the GFP reporter expression data. The combined methodological approach presented here can be adopted to generate expression data for other genes of interest and reporter mice.

• We present an optimized method to systemically characterize gene expression in fluorescent reporter mouse strains with a single cell resolution.
• We methodically combine systemic gene expression profiling with an adjusted mass spectrometry protocol to document acute protein levels in selected organs of interest in mice.

## Graphical abstract

**Figure:**
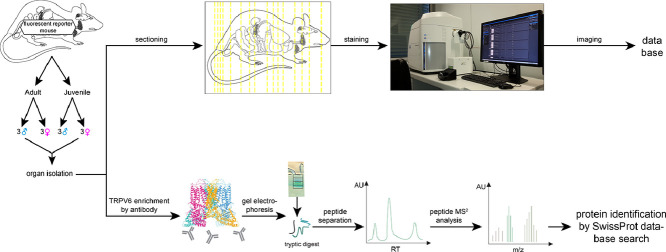

Specifications table

**Table:** 

Subject Area:	Biochemistry, Genetics and Molecular Biology
More specific subject area:	*Genetically modified mouse models*
Method name:	*Combination of immunoprecipitation/mass spectrometry with genetic labeling in mice*
Name and reference of original method:	*1. Wyatt A, Wartenberg P, Candlish M, Krasteva-Christ G, Flockerzi V, Boehm U. Genetic strategies to analyze primary TRP channel-expressing cells in mice.* *Cell Calcium. (2017), 67:91-104.* *2. Fecher-Trost C, Wissenbach U, Beck A, Schalkowsky P, Stoerger C, Doerr J, Dembek A, Simon-Thomas M, Weber A, Wollenberg P, Ruppert T, Middendorff R, Maurer HH, Flockerzi V. The in vivo TRPV6 protein starts at a non-AUG triplet, decoded as methionine, upstream of canonical initiation at AUG. J Biol Chem (2013), 288:16629-16644.* *3. Fecher-Trost C, Lux F, Busch KM, Raza A, Winter M, Hielscher F, Belkacemi T, van der Eerden B, Boehm U, Freichel M, Weissgerber P. Maternal Transient Receptor Potential Vanilloid 6 (Trpv6) is involved in offspring bone development. J Bone Miner Res (2019), 34:699-710.*
Resource availability:	N.A.

## Method details

### Preparation of the 2D expression atlas

**Step 1: Perfusion of animals and dissection of organs**

Material

• fluorescent reporter mice (in our case: TRPV6-IRES-Cre/eR26-τGFP mice, adult and juvenile)
• PBS (3 M NaCl, 161 mM Na_2_HPO_4_*2H_2_O, 39 mM KH_2_PO_4_)
• 4% paraformaldehyde (PFA) in PBS
• anesthesia according to your country's animal laws
• 18% sucrose in PBS
• 0.5 M EDTA

All animals, regardless of age, are transcardially perfused and all organs are removed. All dissected organs are kept in 4% PFA on ice for three hours. The thorax package is stored in a 50 ml falcon tube, the pituitary and trigeminal ganglia in a 1.5 ml reaction tube, while the rest are stored in 15 ml falcon tubes [1], [2].

1. Fill one syringe with PBS and the other with PFA, place the PFA syringe on ice.
2. Anesthetize animals and wait until reaction to a toe pinch is no longer observed before cutting the chest open and exposing the heart. We use a mix of xylazine (32 mg/kg) and ketamine (200 mg/kg).
3. Make a cut in the right atrium, place the needle in the left ventricle and perfuse the animal with PBS (volume depends on the age, until the liver is clear and no more blood comes out of the atrium) followed by ice-cold 4% PFA (60 – 80 ml for adult, 20 – 40 ml for juvenile).
4. Remove the skin on top of the salivary gland (Fig.1 A) and cut out the salivary gland.

**Fig. 1:**
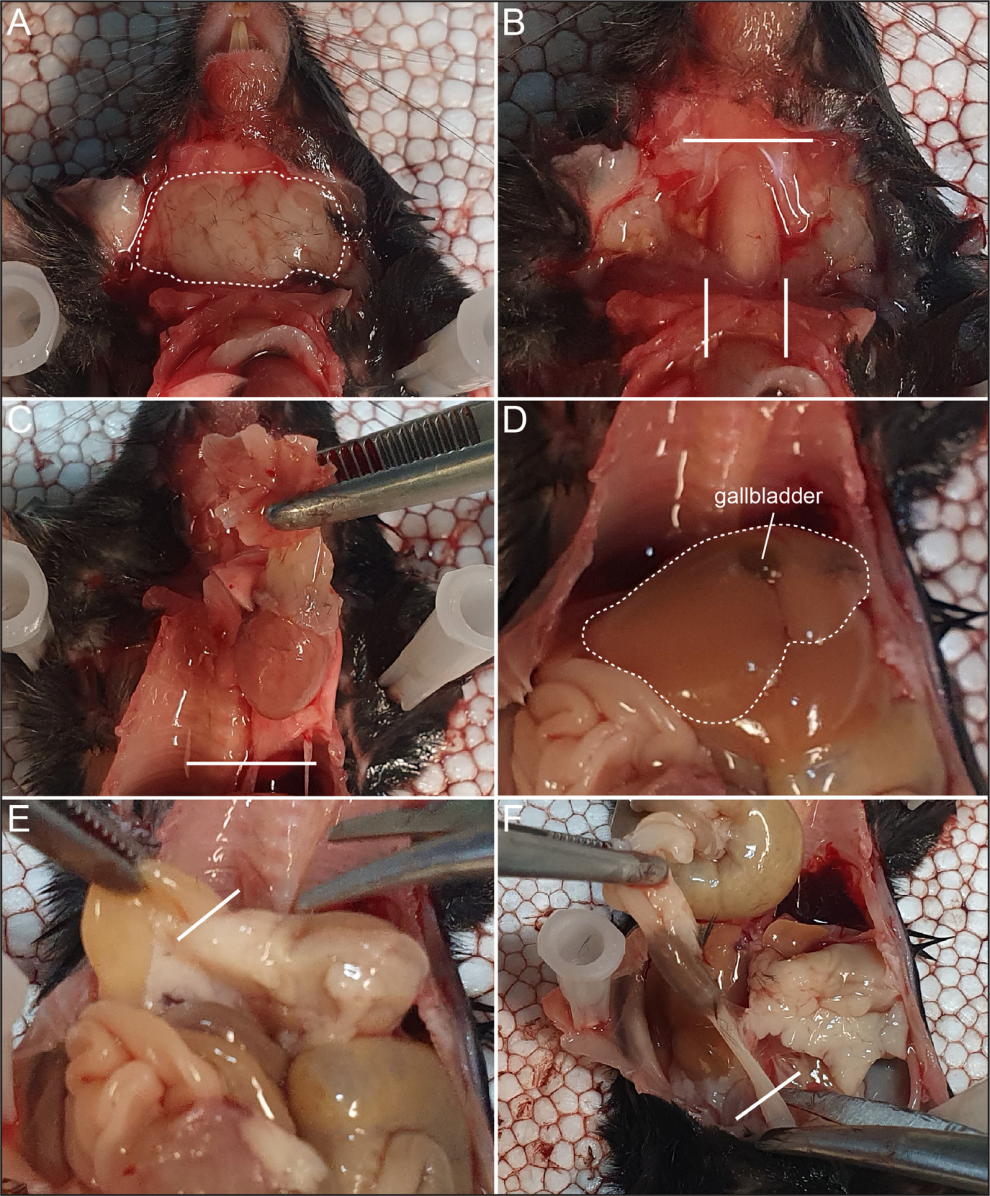
Dissection of thorax and abdominal organs. A, Stitched line represents the salivary gland. B, Solid lines show where to cut to remove the trachea and the thorax package. C, Solid line shows where to cut to remove the thorax package. D, Stitched lines represent the liver and gallbladder. E, Solid line indicates the area to cut the duodenum from the stomach. F, Solid line represents the end of the colon.
5. Cut the trachea close to the tongue (Fig. 1 B) and lift it up. With a blunt edged scissor cut behind the trachea and remove the thymus, heart and lung together with the trachea as one package (Fig. 1 C).
6. Remove a piece of liver including the gallbladder (Fig. 1 D). You only need a small piece of liver including the gallbladder. The rest of the liver can be cut away.
7. Cut the duodenum close to the stomach (Fig. 1 E) and remove the whole intestinal tract, cut at the end of the colon (Fig. 1 F).
8. Cut out a piece of duodenum, jejunum, ileum and colon and remove the cecum (Fig. 2). 0.5 mm in length for each intestinal compartment is sufficient.

**Fig. 2:**
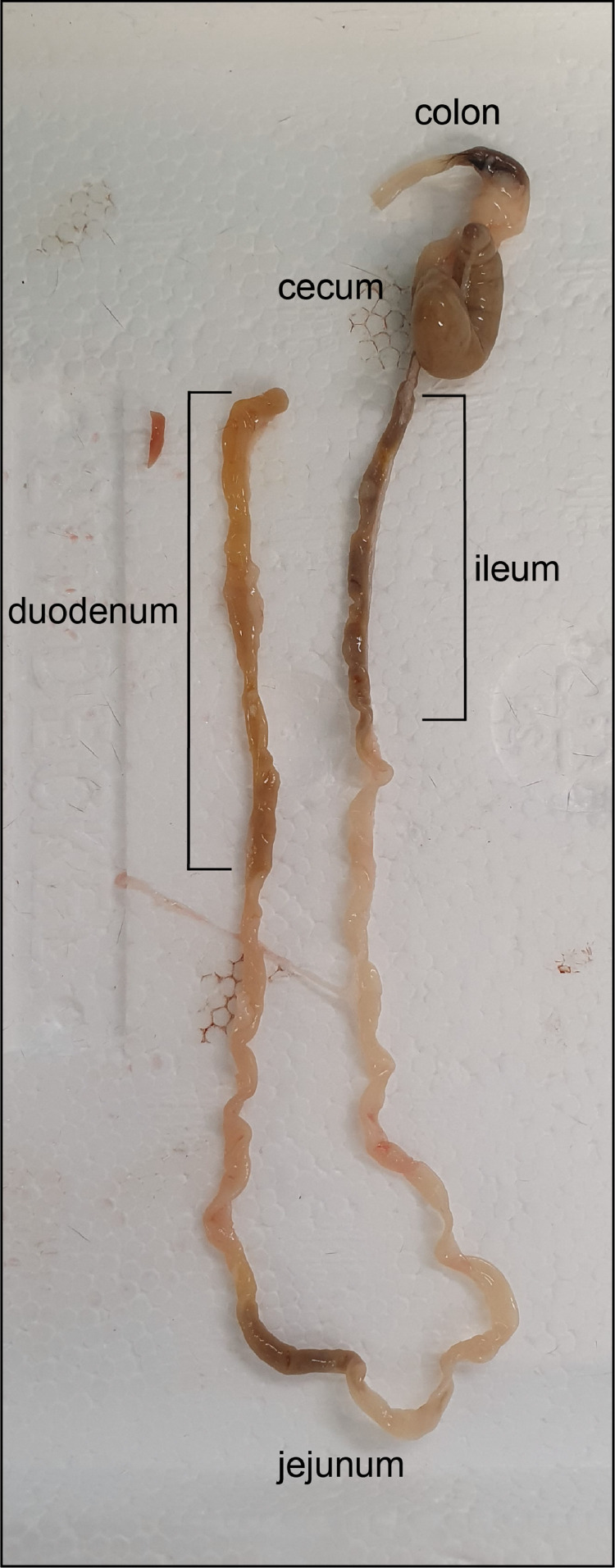
Complete mouse intestine. Different compartments are indicated.
9. Dissect the stomach and cut away the attached pancreas (Fig. 3 A). When removing the pancreas, you can leave the spleen attached (Fig. 3 B).

**Fig. 3:**
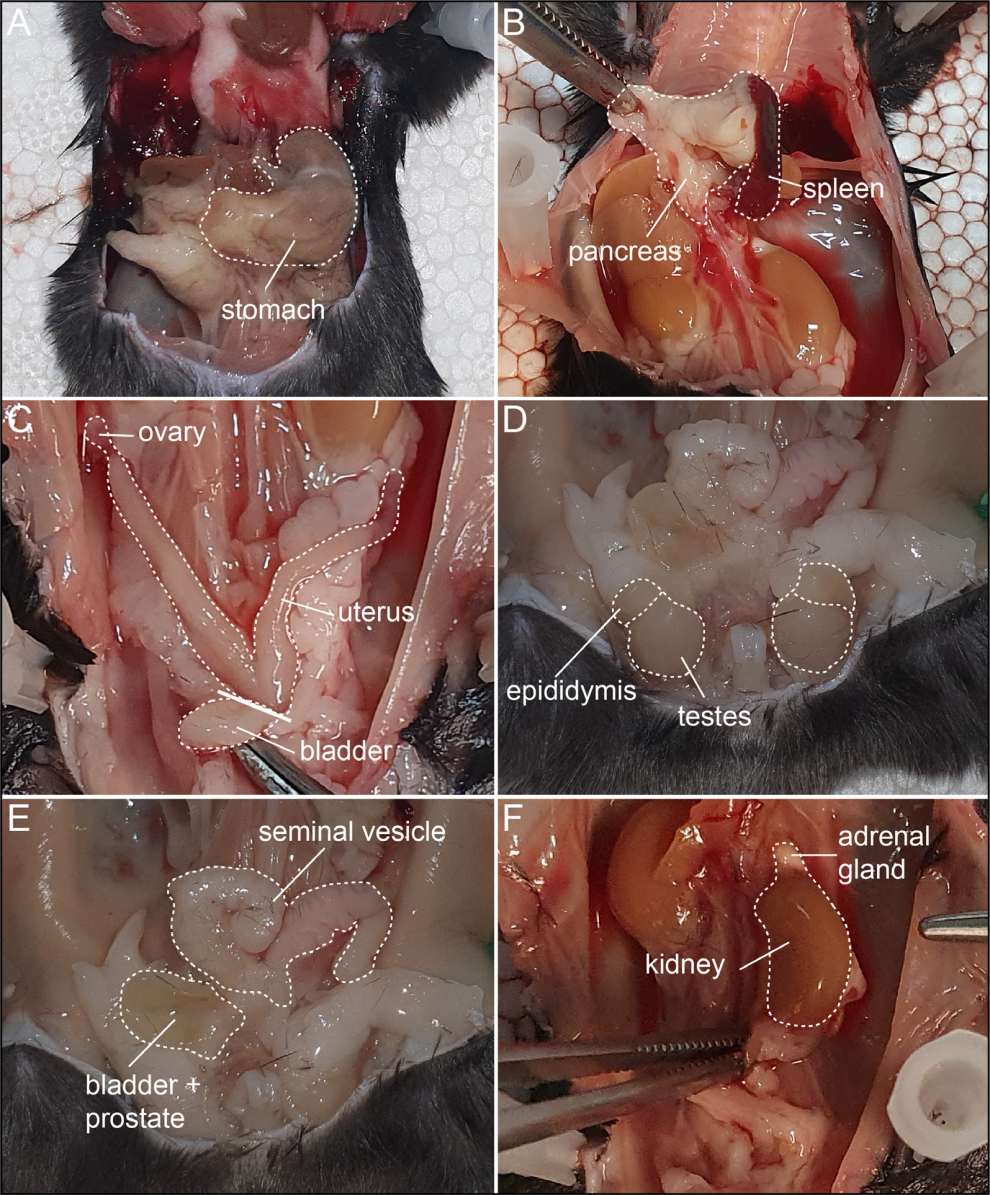
Dissection of the stomach, pancreas, spleen, gonads and urinary system. A, Stitched line indicates the stomach. B, Pancreas and spleen after the removal of the stomach are indicated. C, In females the uterus can be removed with the ovaries attached, then the bladder is removed. D, In males, the testes are removed with the epididymis attached. E, Following removal of the seminal vesicle, the bladder is also dissected together with the prostate. F, Kidney and adrenal gland are indicated by stitched lines.
10. In females, remove the bladder, and subsequently the gonads (uterus with ovaries) (Fig. 3 C).
11. In males, first expose the testes by pushing them out and cut the vas deferens to remove the testes with epididymis (Fig. 3 D). Next, dissect the seminal vesicle and cut the bladder out with the prostate attached (Fig. 3 E).
12. Next, remove the kidney with the adrenal gland attached (Fig. 3 F).
13. Cut the head off at the neck, make a cut through the skin along the midline and fold the skin to the side. Make three cuts into the skull with bone scissors, one along the midline and two on the side (Fig. 4 A) and carefully remove the top of the skull with forceps. Remove the bones on top of the olfactory bulb and lift the brain out of the skull cavity by inserting forceps at the back of the brain under the cerebellum, lifting the brain forward, while flipping it towards the nose. The still attached ocular nerves can be gently pulled loose using forceps, to release the brain (Fig. 4 B).

**Fig. 4:**
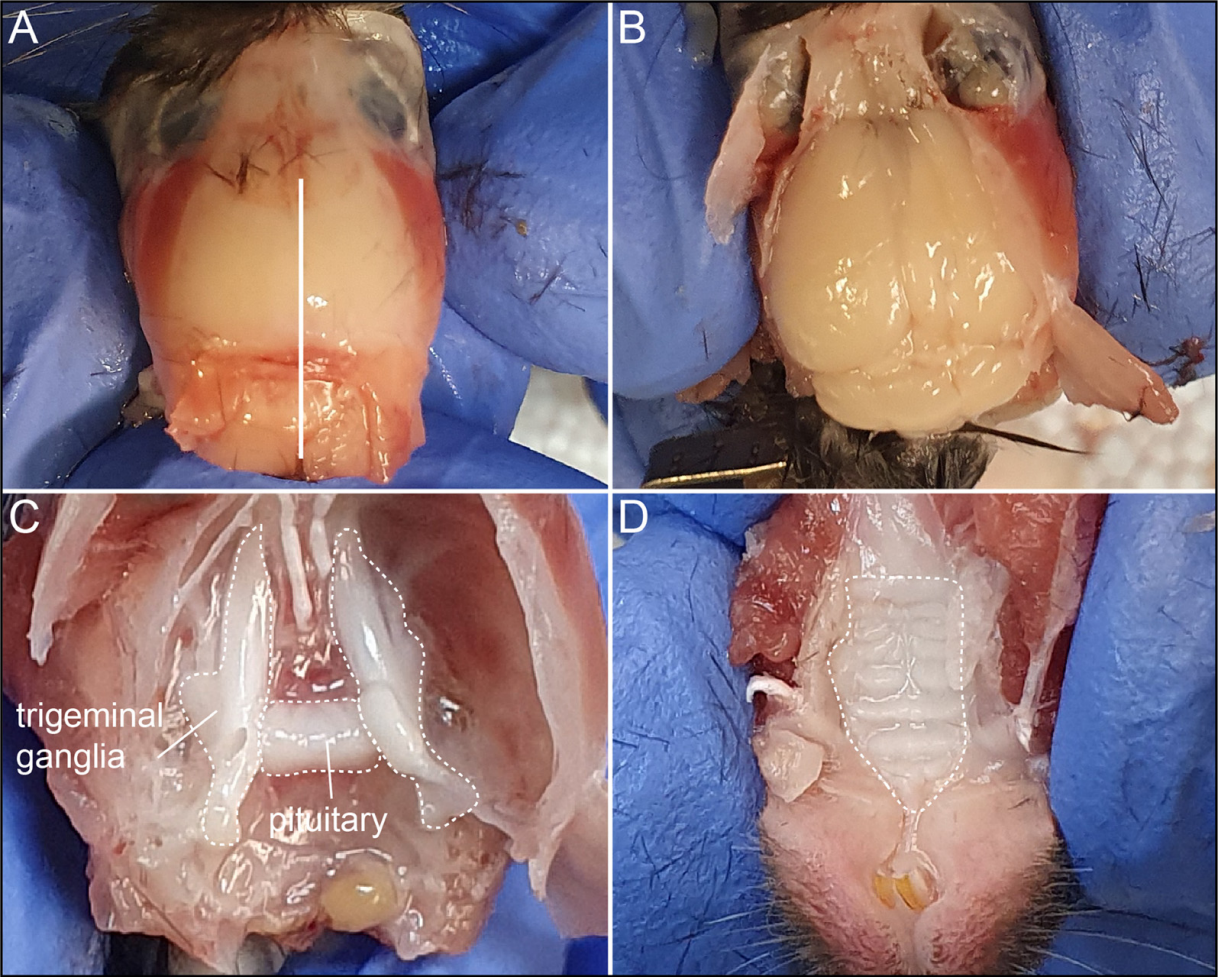
Dissection of the neuronal and olfactory system. A, Solid line indicates midline. B, Removed skull exposes the brain. C, Pituitary and trigeminal ganglia are indicated. D, Hard palate covering the olfactory tissue is indicated.
14. To remove the pituitary gland (Fig. 4 D), gently loosen the pituitary by pushing against it with forceps. You can pick it up with forceps or wash it into a 1.5 ml reaction tube with PFA and a pipette. You can also dissect the attached ganglia by carefully cutting out the marked area in Fig. 4 D with a scalpel. (Note: this method only works when the animal is fixed, in unfixed animals the pituitary will break).
15. To dissect the tongue, first cut the lower jaw away and then cut the tongue at the epiglottal region to remove it.
16. Next, remove the remaining skin from the head and take out the eyes from their sockets then use fine forceps to remove the hard palate (Fig. 4 F) and expose the olfactory tissue.
17. To prepare the nose, remove any remaining skull and as many muscles as you can. Leave the teeth in place until after decalcification.

After three hours post fixation in 4% PFA, change the organs into 18% sucrose and keep them at 4°C until the following day. Do not keep the organs in sucrose longer as it leads to sectioning difficulties. The nose is transferred into 0.5 M EDTA for a 3 to 4 days at 4°C before it is placed in 18% sucrose (in this case for only 3 hours before freezing, see also below).

**Step 2: Organ freezing and preparation of cryosections**

Material

• embedding molds (22 × 22 × 20 mm, Thermo Fischer, # 2219)
• tissue freezing medium, for example OCT (Leica, #14020108926)
• staining glass container (for example: Thermo Fisher #15222349)
• dry ice
• ethanol
• isobutane (Roth, #3927.1)
• glass slides (Superfrost, R. Langenbrinck GmbH, #03-0060 or ThermoFisher, #10149870)
• cryostat with motor, for example CM3050 S from Leica
• blades (Thermo Fischer, #3053835)
• brush

**Step 2.1: Organ freezing**

We suggest incubation of all organs for three hours at room temperature (RT) in OCT before freezing. For this, you can use standard 24 well plates but it is recommended to place each organ in its own well as this improves sectioning results. Before transferring organs from sucrose to OCT, blot the sucrose away using tissue paper and dissect some organs further (see below, Point one). We recommend the isobutane/dry ice method, described below, over other freezing methods as the tissue block freezes more homogeneously and it affords better control over organ orientation.

1. Before placing in OCT wells, try to dissect as much fat away from the organs as possible as fat will interfere with sectioning. Cut the trachea off the thorax package just above the thymus and put these two pieces separately into OCT wells. Separate the pancreas from the spleen and place each organ into a single well. Make a small cut into the stomach and remove the food before placing in the OCT well and try to pump OCT into the heart and stomach by gently squeezing the organ with feather forceps.
2. Our recommended embedding molds have plastic demarcations marking the front of the tissue block. If you are using different ones make sure to mark the front of the tissue block for better orientation and reproducibility of sections. Place the organ in the empty embedding mold in the appropriate orientation (see organ-specific details below) and fill with OCT until the organ is covered. Add an additional 1-2 mm of OCT on top of the organ for cryostat adjustments.
3. Organs are always frozen in the same orientation for reproducibility. The following organs are frozen by placing them at the bottom of the embedding mold:
  a. salivary gland
  b. liver, with gallbladder facing upwards
  c. stomach, with esophagus facing upwards
  d. pancreas
  e. cecum
  f. kidney, with adrenal gland facing the upper edge of the embedding mold
  g. bladder, similar for both male and females, for males keep in mind that the prostate is attached at the lower end of the bladder
  h. gonads
  i. pituitary
4. The rest of the organs are frozen at a 90° angle to the bottom of the embedding mold:
  a. trachea, the end that was close the thymus is facing downwards.
  b. thorax package, with thymus facing upwards.
  c. intestine, you can either freeze each subcompartment in one block if you have multiple animals or the four different parts in one block for each animal. We recommend to note in which corner you placed which tissue piece.
  d. tongue, the tip of the tongue facing upwards. This way you will reach the taste buds early during sectioning.
  e. nose, the tip of the nose is facing upwards.
  f. brain, the bulb is facing upwards and the hypothalamus is facing the front of the embedding mold.
5. Place the staining glass in a second larger container and surround it with dry ice. Fill the glass with isobutane around 0.5 to one cm high. Add ethanol to the dry ice. Make sure that the ethanol level is higher than the isobutane level.

**Step 2.2: Preparation of cryosections**

We recommend to section all organs in series of five. This means that section number two on slide number one is the sixth section in the series. This method was originally established from detailed brain mapping as this way the distance between one section to the next on the same slide is roughly the same as the distance represented from one brain atlas picture to the next. We define the section with the same position on subsequent slides as equal. This way you can do multiple stainings on equal sections. All organs are sectioned with a thickness of 10 µm, with the exception of the brain and pituitary which are sectioned with 14 µm. We highly recommend the use of disposable blades and a brush over the use of normal blades and a glass extender for sectioning.

For a general mapping, staining and imaging of representative sections from one series out of five is enough for each organ.

**Step 3: Staining and imaging of cryosections**

Material

• lambda carrageenan (Sigma, #22049)
• primary antibody (for example chicken anti-GFP, Invitrogen #A10262 or rabbit anti-GFP, Invitrogen #A11122)
• secondary antibody (for example goat anti chicken 488, Invitrogen #A11039 or donkey anti rabbit biotinylated, Vector Labs #VEC-BA-1000 followed by streptavidin Cy5, Jackson Immuno Research #016-170-084)
• Hoechst 33258 (5 mg/ml, Sigma #94003)
• blocking solution (10% donkey serum, 3% bovine serum albumin, 0.03% NaN_3_, 0.3% TritonX-100)
• PBST (PBS with 0.05% Tween-20)
• mounting medium (FluoromountG, SouthernBiotech, # 0100-01)
• cover slips
• staining container (KartellLABWARE #922)
• glass container for washing (ThermoFisher, #10375681)
• fluorescent microscope enabling imaging as tile images (AxioScan from Zeiss in our case)

We use Carrageenan-PBS as a dilution medium for our antibodies. This solution containing the diluted antibodies can be used many times and stored at 4°C, minimizing the amount of antibody required. In combination with our recommended staining container, there is no need for a barrier pen and the sections will not dry out. For rehydration we use 1x PBS and 1x PBST for washing. Stir both PBST and blocking solution for at least 30 min after adding Tween-20 and TritonX-100 respectively.

1. Dissolve 5 g lambda carrageenan and 0.2 g sodium azide in 1 liter 1x PBS. Heat slowly to 40°C. It is important to add the carrageenan first and then heat up. Let it stir between 37°C and 40°C overnight. Do not let the solution get warmer than 40°C. On the following day, filter the carrageenan by gravity through filter paper and store at 4°C. Volumes of up to 1 liter can be made as the buffer is stable at 4°C.
2. Dissolve your antibodies in PBS-Carrageenan and fill the staining container with your antibody solution. Our recommended containers have a capacity of 10 slides and need 38-40 ml of antibody solution to cover the sections. You can reuse these solutions many times.
3. Working solution of our antibodies: 1:1000 for chicken anti-GFP (primary) and 1:500 for goat anti-chicken 488 (secondary). We recommended to titrate the primary to determine the best dilution. We recommend that secondary antibodies are diluted 1:500.
4. After the initial dehydration step do not let the slides dry out at all. We do all washing steps with gentle agitation.

Detailed staining protocol:

a. Take the slides out of -80°C and let them dry for 30 min at room temperature (RT). After this step, slides should not be allowed to dry out.
b. Rehydrate the slides by washing three times for five minutes in PBS.
c. Block the sections in blocking solution for 1 h at room temperature. For our blocking solution, we also use the container system enabling this buffer to be reused multiple times. When staining results worsen or the solution exhibits a color change, the blocking solution should be exchanged.
d. Transfer the slides from the blocking container into the primary antibody container and incubate overnight at 4°C. Different primary antibodies can be mixed in one container and incubated together. If subsequent incubations are instead used, wash then with 1x PBST three times for five minutes with agitation between the incubation steps.
e. In general, always wash your slides at least three times for five minutes at RT with agitation between incubation steps.
f. Incubate your slides in secondary antibody for two hours at RT.
g. Wash three times for five minutes in PBST with agitation.
h. Incubate the slides in Hoechst solution for five to ten minutes at RT when additional nuclear stain is desired.
i. Coverslip all sections with mounting medium.

For imaging, we highly recommend a fluorescent microscope which is capable of producing tile images in order to image the whole, or the majority of the section in one picture to give an overview of each organ. We use the AxioScan slidescanner from Zeiss to automate the imaging process. After imaging we postprocess the pictures and pick out representative images for each organ, sex and age to upload into our database.

### Enrichment of proteins from mouse tissue by antibodies and detection by mass spectrometry

For the enrichment of a protein, specific antibodies can be either covalently immobilized to, for example, tosyl-activated affinity magnetic beads (a), or monoclonal or polyclonal antibodies can be bound to protein A/G magnetic beads (b). The later interaction between antibody and protein A/G magnetic beads is not covalent. The covalent immobilization of antibodies to magnetic beads can be undertaken prior to the planed enrichment experiment and these are stable at 4°C for several months. It is recommended that tissues or cells to be used for the protein enrichment experiment, are collected at an earlier date, snap frozen in liquid nitrogen then stored at -80°C. Frozen or fresh protein lysates are prepared by disruption in the presence of a detergent-containing buffer (e.g RIPA buffer). After ultracentrifugation, the protein lysates are used for incubation with the immobilized antibodies [3], [4].

**Step 1: Covalent coupling of monoclonal- or polyclonal antibodies to tosylated magnetic beads**

Material

• Dynabeads M-280 tosylactivated (ThermoFisher, Invitrogen, No. 142.04)
• magnetic rack (for 1.5 ml reaction tubes, ThermoFisher)
• affinity-purified antibodies (polyclonal antibodies should be purified against their antigenic peptide or protein, monoclonal antibodies from mouse for example can be purified from cell culture medium with protein A/G-agarose. The antibody concentration should be 1 mg/ml in 100 mM HEPES pH 7.6 or in coupling buffer B (see below.) If the concentration is too low or the antibody is not in the appropriate buffer, the solution should be ultrafiltrated and concentrated (for example with Vivaspin MW 50000 or 100000, or Millipore)

Determine the immunoglobulin concentration by absorption measurement at A280 nm: 1.25= 1mg/ml IgG.

Coupling buffers

• buffer B: 0,1 M sodium phosphate pH 7.4: 2.62 g NaH_2_PO_4__*_H_2_O (MW: 137.99 g/mol) and 14.42 g Na_2_HPO_4__*_H_2_O (MW 177.99 g/mol) made to 1 liter
• buffer C: 3M ammonium sulfate in buffer B: 39.64 g (NH_4_)_2_SO_4_ in buffer B made to 1 liter
• buffer D: PBS pH7.4 + 0.5% (w/v) BSA
• buffer E: PBS pH 7.4 + 0.1% BSA, 0.05% NaN_3_
  1. Pipette 165 μl tosyl-activated beads (5 mg) in a 1.5 ml reaction tube, collect the beads with a magnetic rack, discard the supernatant.
  2. Wash the beads once with 1 ml buffer B, vortex, collect the beads in the magnetic rack, discard the supernatant.
  3. Add 100 μg antibody to 100 mM HEPES pH 7.6 to give a total volume of 100 μl.
  4. Add 50 μl buffer B (max. 150 μl volume, note: coupling also works in 150 μl 100 mM HEPES buffer inclusive antibody).
  5. Add 100 μl buffer C.
  6. Vortex, incubate at RT for 18-24 h or 12-18 h at 37°C.
  7. Collect the beads in the magnetic rack, discard the supernatant.
  8. Add 1 ml buffer D for blocking (overnight at 4°C, or 2 h at RT or 1 h at 37°C), collect the beads and discard the supernatant as previously described.
  9. Wash twice with 1 ml buffer E, dissecting the supernatant after each as previously described.
  10. Resuspend antibody beads in 1 ml buffer E (100 μg antibody/1000 μl solution).
  11. Store antibody-coupled beads at 4°C until use (antibody column is stable for at least 3-6 months).

**Step 2: Binding of monoclonal- or polyclonal antibodies to protein A/G magnetic beads**

Material

• Protein A and Protein G magnetic beads (e.g. Pierce Protein A/G Magnetic Beads, ThermoFisher)
• magnetic rack (for 1.5 ml reaction tube, ThermoFisher)
• RIPA buffer: 150 mM NaCl, 50 mM Tris HCl, pH 8.0, 5 mM EDTA, 1% Nonidet P40, 0.1% SDS, 0.5% Na-deoxycholate, pH 7.4 including protease inhibitors (Complete, Mini Protease Inhibitor Cocktail, Roche)
  1. Pipette 50 µl of protein A/G beads into a 1.5 ml reaction tube, collect the beads with a magnetic rack, discard the supernatant.
  2. Wash beads with 1 ml RIPA buffer, collect the beads as previously described and discard the supernatant.
  3. Add 10 µg of monoclonal or polyclonal purified anti-TRPV6 antibodies.
  4. Incubate antibody with magnetic beads for 1 h at room temperature.
  5. Collect the beads with a magnetic rack, discard the supernatant.
  6. Wash antibody beads with 1 ml RIPA buffer, collect the beads with a magnetic rack, discard the supernatant.
  7. Proceed directly with tissue protein lysate incubation (see below) or store antibody-beads at 4°C until use. Do not freeze the antibody-beads.

**Step 3: Preparation of mouse tissue protein lysates**

Material

• 100-500 mg fresh or frozen (-80°C) mouse tissue
• ultracentrifuge (Optima MAX-E, Beckmann Coulter)
• RIPA buffer: 150 mM NaCl, 50 mM Tris HCl, pH 8.0, 5 mM EDTA, 1% Nonidet P40, 0.1% SDS, 0.5% Na-deoxycholate, pH 7.4 including protease inhibitors (Complete, Mini Protease Inhibitor Cocktail, Roche)
• precool buffer at 4°C and add the protease inhibitors always fresh to the RIPA buffer shortly before use
• denaturing buffer: 60 mM Tris HCl, pH 6.8, 4% SDS, 10% glycerol including 0.72 mM β-mercaptoethanol
  1. Collect 100-500 mg mouse tissue (e.g. salivary gland, cecum, pancreas, epididymis, prostate, pregnant uterus) and disrupt tissue manually in 1-5 ml RIPA buffer (4°C) with a Polytron-mixer (Kinematica).
  2. Homogenize tissue in 5-20 ml glass teflon potter (VWR).
  3. Incubate homogenate for 45 min at 4°C with shaking.
  4. Repeat homogenisation with glass teflon potter.
  5. Ultracentrifuge tissue homogenate for 45 min at 100000 xg at 4°C.
  6. Transfer supernatant into fresh falcon tube.
  7. Incubate 250 µl of tosylatedantibody magnetic beads or 10 µg polyclonal/or monoclonal antibody bound to protein A/G magnetic beads with entire tissue protein lysate for 12-16 h at 4°C.
  8. Collect magnetic beads with a magnet rack, discard the supernatant.
  9. Wash the antibody columns five times with 1 ml RIPA buffer (including protease inhibitors) using the magnetic rack.
  10. After the last washing step, remove washing solution from the magnetic beads very carefully.
  11. Elute antibody complex by adding 50 µl denaturing buffer and incubate at 60°C for 20 min.
  12. Store elute at -20°C until gel electrophoresis can be performed.

**Step 4: Gel electrophoresis and sample preparation for mass spectrometry**

Material

• vacuum concentrator Speed Vac Plus SC110A (Savant)
• 4-12% NuPAGE gels and MOPS electrophoresis buffer (ThermoFisher)
• fixation buffer: 40% (v/v) ethanol, 10% (v/v) acetic acid (96%)
• blue silver staining solution: 0.12% (w/v) Coomassie G250 dye, 10% (w/v) ammonium sulfate, 10% (v/v) phosphoric acid and 20% (v/v) methanol
• solution A: 50 mM NH₄HCO₃ (800 mg/200 ml), prepare fresh
• solution B: Solution A and acetonitrile (CH_3_CN) in the ratio 1:1 (25 ml/ 25 ml), prepare fresh
• reducing solution: Solution A and 10 mM dithiothreitol (DTT, 77.5 mg/50 ml), prepare fresh
• alkylating solution: Solution A and 5 mM iodoacetamide (46.5 mg/50 ml) prepare fresh and store in the dark!
• extraction Buffer: 2.5% (v/v) formic acid (FA) in 50% acetonitrile
• MS Buffer: 0.1% (v/v) formic acid in H_2_O
• glass vials (N11, flat, 1.5 ml), glass insert (15 mm tip, 0.2 ml, wide opening, 6 × 31 mm), Bördellk crimp caps (N11, TEF 1.0 mm), all from Macherey-Nagel, Germany

To prepare buffers use ultrapure water (MilliQ) and MS grade quality acetonitrile and chemicals.

1. Load 25 µl eluate (after immunoprecipitation) on NuPAGE 4-12% gradient gels.
2. Let the samples migrate 1-1.5 cm into the gel at 180 V. Stop the electrophoresis.
3 Remove gel from the cassette.
4. Fix the gel for 30 min at RT in fixation buffer.
5. Wash the gel three times for 10 min H_2_O (distilled), use a minimum volume of 100 ml for each washing.
6. Stain the gel for 30 min to 2 h at RT (or overnight) until blue protein staining is visible.
7. Destain gel with H_2_O (distilled) until background becomes clear.
8. Based on the blue stain, cut the protein-containing fragment out with a fresh scalpel and cut it further into three to four pieces and transfer them to separate 1.5 ml Eppendorf tubes. Store gel bands at -20°C until use or proceed with washing.
9. Incubate each gel piece separately with 300 μl solution A for 5-10 minutes at RT while shaking.
10. Remove the solution.
11. Incubate gel pieces with 300 μl solution B for 5-10 minutes while shaking.
12. Repeat alternate washing with buffers A and B again.
13. Add 300 μl reducing solution and incubate for 30 minutes at 56°C.
14. Centrifuge (1000 xg, RT) and remove solution again.
15. Add 300 μl alkylating solution and incubate 30 minutes at RT in the dark.
16. Centrifuge (1000 xg, RT) and remove solution.
17. Repeat steps 9-12.
18. Evaporate gel pieces in the vacuum centrifuge until they are completely dry. The gel pieces will turn whitish/milky.
19. Depending on the size of the gel piece add 15-25 μl porcine sequencing grade trypsin (Promega, reconstitute 20 µg trypsin in 1 ml buffer A); use enough trypsin solution to rehydrate and cover the gel piece. The gel piece should not swim in trypsin solution. Incubate proteins overnight at 37°C.
20. Add 50 µl extraction buffer and place tubes in the ultrasonic bath for 15 minutes.
21. Centrifuge gel pieces (15000 rpm for 5 seconds).
22. Remove the supernatant and transfer it to a new tube.
23. Repeat steps 20-22, then incubate for 15 min in ultrasonic bath and combine the supernatants.
24. Place the tubes in the Speedvac for 1 hour until liquid is evaporated.
25. Add 20 μl MS buffer.
26. Put the tubes in the ultrasonic bath for 10 minutes.
27. Centrifuge at 15000 rpm for 5 minutes.
28. Transfer 18 μl of the supernatant into the glass vial with inlet and close septum.

**Step 5: Nano-LC-ESI-MS/MS measurements and raw data analysis**

Material

• Nano LC-ESI-MS/MS unit: Ultimate 3000 RSLC nano system coupled, LTQ Orbitrap Velos Pro, Ultimate 3000 RS Autosampler (ThermoScientific, TF, Dreieich, Germany)
• trap column (C18, inner diameter 100 µm, length 2 cm, Acclaim PepMap, ThermoScientific), reversed phase column (nano viper Acclaim PepMap capillary pre column, inner diameter 75 µm, length 50 cm, ThermoScientific)
• Nano LC buffer A (water and 0.1% formic acid)
• Nano LC buffer B (90% acetonitrile and 0.1% formic acid)
• coated silica electrospray emitter (PicoTipEmitter, 15 µm, New Objective, USA)
  1. Inject 6 µl of the tryptic peptide extracts generated in step 4 into nanoflow LC-HR-MS/MS system.
  2. Collect and separate tryptic peptides on C18 trap and separation column at a flow rate of 200 nl/min and develop a gradient with Nano LC buffer A and B. Use gradient 1 (4 - 55% buffer B in 30 min; 55 - 90% B in 6 min) or gradient 2 (4 - 55% Buffer B in 56 min; 55 - 90% B in 7 min) for separation. The effluent of the chromatography is directly sprayed into the mass spectrometer through a coated silica electrospray emitter and ionized at 2.2 kV.
  3. Acquire MS spectra in a data-dependent mode. For the collision induced dissociation (CID) MS/MS top^10^ method (gradient 1 and 2) acquire full scan MS spectra (m/z 300 – 1700) in the Orbitrap analyzer using a target value of 10^6^. The 10 most intense peptide ions with charge states >2 are fragmented in the high-pressure linear ion trap by low-energy CID with normalized collision energy of 35%.
  4. For high energy collision dissociation (HCD) top3 method record full scan MS spectra (m/z 300 – 1700) in the orbitrap analyzer with resolution of r = 60000. The 3 most intense peptide ions with charge states >2 are sequentially isolated and fragmented in the HCD chamber with a normalized collision energy of 30%. The resulting fragments are detected in the orbitrap system with a resolution of r = 7500.
  5. Use the MASCOT algorithm for the analysis of fragmented peptides (version 2.4.0, Matrix Science, London) and Proteome Discoverer 1.4 software (ThermoScientific). Load raw files into proteome discoverer.
  6. Match peptides and fragments with the following parameters: SwissProt database (version 2018_05, species mouse, number of protein entries 16.992), allow Mascot searches with a fragment ion mass tolerance of 0.50 Da and a parent ion tolerance of 7.0 PPM, use tryptic digest, and allow for up to two missed cleavage sites. Carbamidomethylation of cysteine is set as a fixed modification and deamidation of asparagine and glutamine, acetylation of protein N-terminus, lysine, and oxidation of methionine are set as variable modifications.

## Declaration of Competing Interest

The authors declare that they have no known competing financial interests or personal relationships that could have appeared to influence the work reported in this paper.
